# Endothelial glucocorticoid receptor promoter methylation according to dexamethasone sensitivity

**DOI:** 10.1530/JME-15-0124

**Published:** 2015-08-04

**Authors:** Eugenia Mata-Greenwood, P Naomi Jackson, William J Pearce, Lubo Zhang

**Affiliations:** Divisions of Pharmacology and Physiology, Department of Basic Sciences, School of Medicine, Center for Perinatal Biology, Medical Center, Loma Linda University, Room A572, 11234 Anderson Street, Loma Linda, CA 92350, USA

**Keywords:** glucocorticoids, endothelium, methylation, 5′UTR mRNA isoform

## Abstract

We have previously shown that *in vitro* sensitivity to dexamethasone (DEX) stimulation in human endothelial cells is positively regulated by the glucocorticoid receptor (*NR3C1, GR*). The present study determined the role of differential *GR* transcriptional regulation in glucocorticoid sensitivity. We studied 25 human umbilical vein endothelial cells (HUVECs) that had been previously characterized as DEX-sensitive (*n* = 15), or resistant (*n* = 10). Real-time PCR analysis of *GR* 5′UTR mRNA isoforms showed that all HUVECs expressed isoforms 1B, 1C, 1D, 1F, and 1H, and isoforms 1B and 1C were predominantly expressed. DEX-resistant cells expressed higher basal levels of the 5′UTR mRNA isoforms 1C and 1D, but lower levels of the 5′UTR mRNA isoform 1F than DEX-sensitive cells. DEX treatment significantly decreased *GR*α and *GR*-1C mRNA isoform expression in DEX-resistant cells only. Reporter luciferase assays indicated that differential *GR* mRNA isoform expression was not due to differential promoter usage between DEX-sensitive and DEX-resistant cells. Analysis of promoter methylation, however, showed that DEX-sensitive cells have higher methylation levels of promoter 1D and lower methylation levels of promoter 1F than DEX-resistant cells. Treatment with 5-aza-2-deoxycytidine abolished the differential 5′UTR mRNA isoform expression between DEX-sensitive and DEX-resistant cells. Finally, both *GR*α overexpression and 5-aza-2-deoxycytidine treatment eliminated the differences between sensitivity groups to DEX-mediated downregulation of endothelial nitric oxide synthase (*NOS3*), and upregulation of plasminogen activator inhibitor 1 (*SERPINE1*). In sum, human endothelial *GR* 5′UTR mRNA expression is regulated by promoter methylation with DEX-sensitive and DEX-resistant cells having different *GR* promoter methylation patterns.

## Introduction

Glucocorticoids are therapeutic agents used for reducing inflammation via targeting the glucocorticoid receptor (*NR3C1, GR*) of immune cells (Auphan *et al.* 1995, Barnes 1998). However, glucocorticoids also target the GR present in various other tissues, causing unwanted side effects. In the cardiovascular system, glucocorticoids induce short-term side effects that include hypertension, dyslipidemia and thrombosis. Chronic synthetic glucocorticoid-therapy has been associated with endothelial dysfunction and increased risk of cardiovascular events such as myocardial infarction (Yang & Zhang 2004, Walker 2007, Hadoke *et al.* 2009, Huang & Glass 2010, Kadmiel & Cidlowski 2013). These adverse events are mediated in part by glucocorticoid-dependent down-regulation of endothelial nitric oxide synthase (*NOS3*) and upregulation of plasminogen activator inhibitor 1 (*SERPINE1*) (Wallerath *et al.* 2004, Tamura *et al.* 2015). Of importance, human studies have revealed significant human variability in response to both endogenous (cortisol) and synthetic glucocorticoids (Ito *et al.* 2006, Kino 2007), but the mechanisms remain undetermined.

Glucocorticoids mediate their biological effects by the ubiquitously expressed GR (Bamberger *et al.* 1996, Adcock *et al.* 2004, Heitzer *et al.* 2007). Alternative splicing produces 3′UTR mRNA isoforms *GRα, GRβ* and *GRP*. GRα protein is the biologically relevant isoform, capable of binding ligand that regulates transcription and stability of target genes. GRβ is localized in the nucleus and has a dominant negative effect on GRα through the formation of GRα/GRβ heterodimers. GRP has been reported to have both synergistic and antagonistic effects on GRα (Kassel & Herrlich 2007, Biddie *et al.* 2012). Previously, we have shown that human endothelial sensitivity to dexamethasone (DEX) varied according to the levels of GRα protein degradation (Mata-Greenwood *et al.* 2013). Human umbilical vein endothelial cells (HUVECs) that were resistant to *in vitro* stimulation by DEX had an increased expression of the E3 ubiquitin ligase gene BCL2-athanogene 1 (*BAG1*) and subsequent GR protein ubiquitination and proteasomal degradation. It was found that the basal protein levels of GRα correlated with the endothelial expression of key genes, such as *NOS3* and *SERPINE1* (Mata-Greenwood *et al.* 2013). However, the transcriptional regulation of GR expression in human endothelial cells and its role in endothelial glucocorticoid sensitivity have not been well defined, and warrant further exploration.

*GR* mRNA expression is regulated by complex mechanisms that include transcriptional and epigenetic modification of *GR* promoters (Presul *et al.* 2007, Turner *et al.* 2010, Cao-Lei *et al.* 2011). The *GR* gene (*NR3C1*) contains at least nine 5′UTR first exons that are spliced to the common exon 2. The alternative first exons are divided into two promoter regions: a distal region located more than 30 kb upstream that contains exons 1A and 1I, and a proximal region located 5 kb upstream of the translation start site that contains exons 1B, 1C, 1D, 1E, 1F, 1H, and 1J. Each first exon is regulated by its own promoter that binds specific transcription factor complexes (Bockmuhl *et al.* 2011, Cao-Lei *et al.* 2011). Therefore, the transcription of the *GR* gene is regulated by complex promoter regions containing multiple transcription start sites. Furthermore, both proximal and distal *GR* promoters are embedded in a CpG island that is mostly methylation-free and therefore can be targeted for methylation. Recent human and rodent studies have shown tissue and disease specific expression of untranslated first exons, due in part to changes in *GR* promoter methylation (Turner *et al.* 2006, 2008, 2010, Bockmuhl *et al.* 2011). These studies have shown that the various *GR* promoters allow for tissue-specific mechanisms to control and adapt *GR* expression levels according to various developmental and pathophysiological stages.

Previous studies have shown that rodent heart and aortic tissue express significant levels of *GR* transcripts (Presul *et al.* 2007, Xiong & Zhang 2013), but the transcriptional regulation of *GR* in specific cardiovascular cells (i.e., endothelial, smooth muscle, cardiomyocytes) remains unknown. Therefore, the main objective of the present study is to uncover the mechanisms that regulate the expression of *GR* mRNA isoforms in human endothelial cells that have been previously characterized as DEX-sensitive or DEX-resistant. We find that DNA methylation plays an important role in the fine-tuning determination of *GR* mRNA isoform expression in human endothelial cells. Our study provides novel insights into the upstream mechanisms that alter *GR* expression in the cardiovascular system and suggest that future use of epigenetic markers will aid in identifying glucocorticoid responsive individuals.

## Materials and methods

### Subjects and cell culture

HUVECs isolated from 25 healthy term pregnancies were selected from a previous cohort study of 42 subjects (Mata-Greenwood *et al.* 2013) to further characterize the transcriptional regulation of GR in human endothelial cells. These HUVECs were previously characterized for endothelial cell purity and *in vitro* response to DEX (Mata-Greenwood *et al.* 2013). The study subject characteristics are summarized in Supplementary Table 1, see section on supplementary data given at the end of this article. The study was approved by the IRBs of the Loma Linda University and the University of California at San Diego. HUVECs were cultured using ECM media (ScienCell, Carlsbad, CA, USA). All assays were performed between passages 4 and 6. To study inter-individual differences in response to glucocorticoids, we stimulated confluent and quiescent HUVECs with DEX using DMSO as a solvent (final concentration of 0.05%). This synthetic glucocorticoid was chosen for its stability in the presence of cellular 11-β-dehydrogenase, and for its specificity for GRα binding with respect to other nuclear receptors, including the mineralocorticoid receptor (Wenting-van Wijk *et al.* 1999). To starve HUVECs, we used M199 (Sigma–Aldrich) supplemented with 0.95 mM HEPES, 0.1% BSA, 1% antibiotics and 1% fetal bovine serum (FBS). The acute monocytic leukemia cell line THP-1 from Sigma–Aldrich was used as a positive control as it is known to express all *GR* mRNA isoforms (Steer *et al.* 2000). THP-1 cells were grown in DMEM with 1% antibiotics and 10% FBS.

### RNA extraction and real-time PCR

Total RNA was extracted with *Tri*ZOL-RNEasy kits (Invitrogen), quantified, and stored at −80 °C until analysis. The total RNA (1 µg) was reverse transcribed and real time PCR was performed for each sample in triplicate as previously described (Goyal *et al.* 2014). Taqman assays were obtained from Life Technologies for *GR* mRNA isoforms 1B (Hs01005211_m1), 1C (Hs01010775_m1), 1D (Hs03666144_m1) and the housekeeping gene 18S (Hs99999901_s1), and these were run according to the manufacturer’s recommendations using a Probe Quantitect PCR mix (Qiagen). Designed primers for SYBR green PCR analysis of the remaining *GR* mRNA isoforms and the housekeeping gene 18S, together with PCR conditions and accession numbers, are shown in Supplementary Table 2, see section on supplementary data given at the end of this article. SYBR green PCR was performed using Quantitect SYBR green PCR kit (Qiagen) using a 2.0 Lightcycler amplifier (Roche). PCR products were purified, sequenced, and used to obtain standard curves for each mRNA isoform. Extrapolation of unknowns from the standard curve was performed using Prism 3 (GraphPad Software, San Diego, CA, USA), predicting unknowns from the standard curve *C*_t_ values. Data is presented as fg mRNA/ng 18S. Alternatively, the percentages of each *GR* mRNA isoform were estimated for each subject as mRNA isoform/sum of all mRNA isoforms × 100.

### SDS–PAGE and immunoblotting

Western blotting was performed as previously described (Mata-Greenwood *et al.* 2010). Briefly, protein extracts (30 µg) were prepared in a cold lysis buffer, heat denatured in a Laemmli buffer, separated on SDS-PAGE, and transferred to PVDF membranes. Membranes were blocked in 5% non-fat dried milk in 0.05% TBST for 1 h, and then probed in primary rabbit anti-GR (Santa Cruz Biotechnologies), monoclonal anti-BAG1 (Santa Cruz Biotechnologies), monoclonal anti-HSP90 (BD Biosciences, San Jose, CA, USA), and rabbit anti-FKBP51 (Stressmarq, Victoria, BC, Canada), diluted in blocking buffer (1 µg/ml) overnight at 4 °C. After three 10 min washes with TBST, the membranes were incubated with secondary antibodies that were diluted at 1:2000. Bound antibodies were visualized using the chemiluminscence substrate (ThermoFisher Scientific, Carlsbad, CA, USA). The membranes were then probed with monoclonal anti-β-Actin (Ambion, Austin, TX, USA). Data is presented as protein levels relative to β-Actin levels.

### Cloning of *GR* promoters

*GR* promoters B, C, D, F, and H were cloned into the firefly luciferase-pGL3 reporter vectors (Promega), as previously described (Mata-Greenwood *et al.* 2010). *GR* promoter regions were selected to include putative essential DNA elements such as initiator (Inr) and downstream promoters (DPE). Briefly, 500 ng of HUVEC DNA (from specific donors) was used to amplify *GR* promoter regions using primers that contained specific restriction enzyme sites (Supplementary Table 3, see section on supplementary data given at the end of this article), followed by ligation into the basic luciferase pGL3 vector. The non-methylated *GR* promoter inserts were confirmed by DNA sequencing. Vector clones containing WT *GR* promoters were used.

### Cell transfection and luciferase assays

Transfection of plasmid DNA was performed with the aid of HD Xtreme transfection reagent (Roche) as previously described (Mata-Greenwood *et al.* 2013). Briefly, confluent cells were transfected with the *GR* promoter/pGL3-luciferase constructs, using a 1:1 complex of DNA:Xtreme reagent according to the manufacturer’s protocol. TK-Renilla luciferase vector (Promega Corp.) was used as the internal control. The transfection was carried out at 37 °C for 6 h. The cells were allowed to recover in a complete culture medium for 18–20 h, and then treated with starvation media with or without DEX (0.2 and 1 µM) for another 24 h, before adding passive lysis. Firefly and Renilla luciferase activities were measured using a dual-reporter assay kit (Promega) according to the manufacturer’s protocol. Relative luciferase values were calculated as a ratio of firefly/renilla luciferase activities. Each treatment was tested in quadruplicates and averages per sample were used to calculate group averages and standard errors.

### Methyl-DNA immunoprecipitation

Genomic DNA was isolated from confluent and quiescent HUVECs using the Wizard genomic DNA isolation kit (Promega). Methyl-DNA immunoprecipitation (MeDIP) was performed with the aid of a commercial kit according to the manufacturer’s protocol (Active Motif, Carlsbad, CA, USA). In brief, 20 µg of DNA was sheared into ~ 200–400 bp fragments and immunoprecipitated with a 5-methylcytosine-specific antibody at 4 °C overnight. Immunoprecipitated DNA was bound to magnetic beads, washed and eluted. Input (sheared DNA) and immunoprecipitated DNA (1 µl) was used to amplify specific promoter regions from the *GR* gene using SYBR green PCR, as previously described. MeDIP real-time PCR primers are shown in Supplementary Table 4, see section on supplementary data given at the end of this article. Promoter methylation levels were estimated as immunoprecipitated DNA/total (input) DNA × 100.

### Bisulfite-converted DNA sequencing

To determine the methylation status of *GR* promoters, DNA samples were treated with bisulfite using the epiTect Bisulfite kit (Qiagen), according to the manufacturer’s instructions. Converted DNA was amplified using primers directed to bisulfite modified genomic DNA (Supplementary Table 4). Amplification conditions were as follows: 94 °C for 30 s, 40 °C for 30 s, and 72 °C for 40 sX40 cycles. Resulting amplicons were cloned into pCR4-TOPO sequencing vectors (Invitrogen) for automated fluorescent sequencing. Data were analyzed using the CLC Software, and clones showing <90% conversion or identified as clonal were not included in further analysis. The methylation levels for each subject were estimated as the methylated clones/#total clones sequenced × 100. A minimum of ten clones per amplicon were analyzed by sequencing. Averages and standard errors were then calculated for each sensitivity group.

### Overexpression of *GRα*

Mammalian expressing vectors for the promoter-less full length *GR-1Cα* (NM_000176.2) were a gift from Dr John A. Cidlowski (NIEHS, Research Triangle, NC, USA). *GR* over-expression was achieved by transfecting log-phase proliferating HUVECs with 1 µg DNA/six well using the Xtreme transfection reagent as previously described. After 18 h of recovery, HUVECs were treated with DEX (1 µM) or not for an additional 24 h before analysis. Overexpression of *GRα* was confirmed by real-time PCR and immunoblotting.

### Global demethylation assays

To induce global demethylation, we utilized a 5 µM dose of 5′-aza-2′-deoxycitidine (AZA) in 50% confluent cells. After 24 h of AZA treatment, cells were treated with both AZA and DEX (1 µM) or solvent for another 24 h before harvesting total RNA. DNA demethylation was confirmed by *GR* promoter D methylation levels of < 1%.

### Procoagulant activity assay

The procoagulant activity of HUVECs was tested by a one-step recalcification time test, also known as the activated partial thromboplastin time (aPTT) as previously described (Mata-Greenwood *et al.* 2013). As discussed, 70% confluent HUVECs were transfected with *GR*α-expressing vectors, and 18 h after were treated with or without DEX (1 µM) for an additional 24 h. Cells were then washed, trypsinized, and resuspended in PBS at a concentration of one million cells per ml PBS. A 1:1 solution was prepared with cells and fresh plasma (collected from one single donor and anticoagulated with 3.8% sodium citrate, 1:9, v/v) and incubated for 180 s at 37 °C. After the addition of preheated CaCl_2_, the time to fibrin strand generation was recorded by a BCS XP hemostatic analyzer (Siemens, Munich, Germany).

### Statistical analysis

Data are presented as means±s.e.m. Each individual variable was analyzed via ANOVA followed by *post-hoc* least significant difference (LSD) analysis to determine differences between sensitivity groups and between treatment groups. Homogeneity of variances was confirmed (*P*>0.05) using the Levene’s Test of Equality of Error Variance. For data sets with unequal variances (*P*<0.05), the data were log transformed and all further statistics were performed on the transformed data. For graphical presentation, the anti-logs of the mean log values were calculated and plotted. In all cases, statistical power was >0.08. A *P* value of <0.05 was regarded as significant. All statistical analysis was performed using SPSS, version 22.

## Results

### Differential *GR* mRNA isoform expression in HUVECs according to DEX-sensitivity

**Figure 1:**
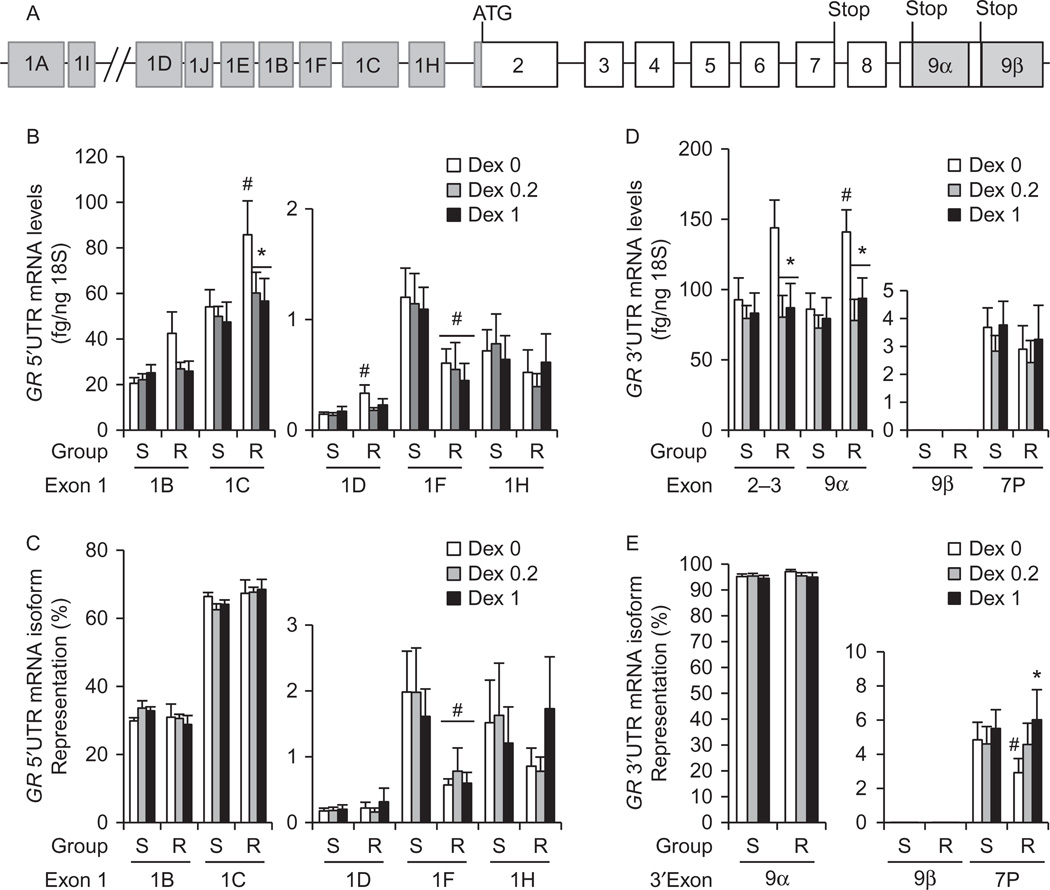
Basal and DEX-stimulated expression of *GR* mRNA transcripts in DEX-sensitive (S) and DEX-resistant (R) HUVECs. (A) Structure of the human *glucocorticoid receptor* gene (NR3C1) that includes 9 exons 1 and 3 stop signals that result in both 5′UTR and 3′UTR mRNA isoforms. (B, C, D and E) Confluent and quiescent HUVECs were treated with solvent, DEX 0.2, and DEX1 µM for 24 h and *GR* mRNA isoforms were quantified by real-time PCR as described under methods. (B) Basal and DEX-stimulated expression levels of *GR* 5′UTR mRNA isoforms (1B, 1C, 1D, 1F, and 1H) expressed as fg *GR* isoform/ng *18S* RNA. (C) Expression of *GR* 5′UTR mRNA isoforms as percent of total *GR* mRNA. (D) Basal and DEX-stimulated expression of *GR* 3′UTR mRNA isoforms (*GRα, GRP* and *GRβ*) as fg GR isoform/ng *18S* RNA; (E) Expression of *GR* 3′UTR mRNA isoforms as percent of total GR mRNA. THP1 monocytoid cells were used as a positive control to verify that HUVECs do not express isoforms 1A, 1E, 1I and 1J. Bars represent the average±s.e.m. (*n* = 15 DEX-sensitive and 10 DEX-resistant). *P<0.05 DEX-treated vs untreated; ^#^*P*<0.05 DEX-resistant cells vs DEX-sensitive cells.

We have previously characterized a small cohort of 42 HUVECs obtained from healthy pregnancies for DEX-sensitivity. DEX-sensitive HUVECs expressed significantly higher levels of GRα protein than DEX-resistant HUVECs (Mata-Greenwood *et al.* 2013). Analysis of study group characteristics indicated that the pre-pregnancy maternal BMI and average systolic blood pressure were significantly higher in DEX-sensitive HUVECs compared to those of DEX-resistant cells (Supplementary Table 1), but the remaining parameters were not significantly different. To further understand the transcriptional regulation of *GR* in endothelial cells, and the mechanisms leading to the differences in *GR* expression between DEX-sensitive and DEX-resistant HUVECs, we analyzed the expression of 5′UTR mRNA isoform transcripts in 25 HUVECs (15 DEX-sensitive and 10 DEX-resistant). HUVECs expressed *GR* proximal mRNA isoforms 1B, 1C, 1D, 1F, and 1H, but did not express the distal isoforms 1A and 1I, or the proximal isoforms 1E and 1J (Fig. 1A and B). DEX-resistant HUVECs expressed significantly higher basal, but similar DEX-stimulated, *GR* mRNA isoform 1C levels (Fig. 1B); and lower percentages of mRNA isoform 1F levels (Fig. 1C) than DEX-sensitive cells. DEX treatment significantly decreased the expression of mRNA isoform 1C in DEX-resistant cells only (Fig. 1B). The percentages of *GR* 5′UTR mRNA isoforms in all HUVECs were ~67% (1C), ~30% (1B), ~2% (1F), ~1% (1H), and ~0.2% (1D) (Fig. 1). DEX treatment did not significantly alter the percentages of *GR* 5′UTR mRNA isoforms in either DEX-sensitive or DEX-resistant cells (Fig. 1C).

We also analyzed the expression profile of *GR* 3′UTR mRNA isoforms *GRα, GRβ* and *GRP*. *GRα* represented ~ 96% of all 3′UTR mRNA isoforms, followed by *GRP* (4%), and *GRβ* expression was slight (<0.0006%) in all HUVECs (Fig. 1E). DEX-resistant HUVECs expressed significantly higher basal levels of *GRα* than DEX-sensitive HUVECs (Fig. 1D) that correlate with higher basal levels of the predominant 5′UTR mRNA isoform 1C (Fig. 1B). Importantly, DEX treatment decreased the expression of *GRα* (Fig. 1D) and increased the percentage of *GRP* (Fig. 1E) in DEX-resistant cells only. Lastly, there were no significant differences in the levels of total *GR* mRNA (measured by exons 2–3) between the sensitivity groups. However, DEX downregulated total *GR* mRNA in DEX-resistant cells only (Fig. 1D).

### Role of *GRα* overexpression on its own transcriptional regulation and endothelial cell phenotype

**Figure 2:**
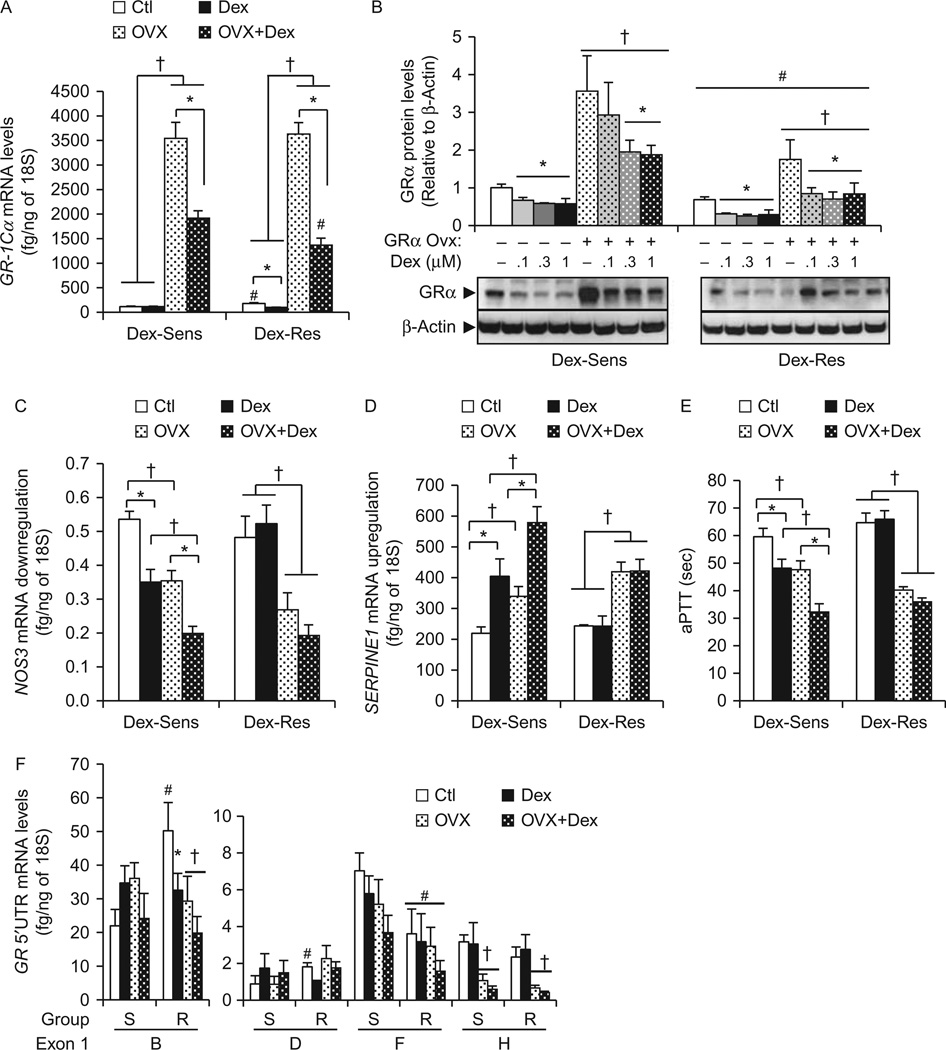
Role of GRα overexpression in endothelial phenotype, expression autoregulation, and response to DEX. HUVECs were transfected with a promoter-less *GR-1Cα* expressing vector, then treated for 24 h with or without DEX (1 µM). Total RNA was extracted, reverse-transcribed and quantified by real-time PCR. (A and B) *GRα* overexpression in HUVECs upregulated both mRNA (A) and protein (B) levels in both DEX-sensitive (*n* = 5) and DEX-resistant (*n* = 5) HUVECs. (C, D and E) Effect of GRα overexpression on *NOS3* downregulation (C), *SERPINE1* upregulation (D) and activated partial thromboplastin time, aPTT (E). (F) Effect of *GRα* overexpression on *GR* 5′UTR mRNA isoform levels. Bars represent the average±s.e.m. (*n* = 5 DEX-sensitive and 5 DEX-resistant). *P<0.05 DEX-treated vs untreated; ^#^*P*<0.05 DEX-resistant vs DEX-sensitive cells; ^†^*P*<0.05 basal vs GRα overexpression.

To clarify the role of differential *GR* 5′UTR mRNA isoform levels between sensitivity groups on endothelial phenotype, we overexpressed *GRα* in both DEX-sensitive and DEX-resistant HUVECs, using a promoter-less *GR-1Cα*-expressing vector. We hypothesized that GRα overexpression would increase sensitivity to DEX in any HUVEC tested. To validate our methods, we first showed our protocol significantly increased *GR-1Cα* mRNA levels by 31-fold and 20-fold, and GRα protein levels by 3.5-fold and 2.4-fold in DEX-sensitive and DEX-resistant cells respectively (Fig. 2A and B). DEX-treatment significantly decreased the levels of overexpressed *GRα* mRNA and protein in both sensitivity groups (Fig. 2A), suggesting DEX-dependent post-transcriptional and post-translational mechanisms of *GR* regulation (Pratt *et al.* 2006, Turner *et al.* 2010). However, our overexpression system had a smaller effect on GR protein levels (two- to fourfold increase) compared to *GR* mRNA (20- to 31-fold increase) levels that is most likely due to the reported GR protein degradation by the proteasome (Wallace *et al.* 2010, Mata-Greenwood *et al.* 2013). Of interest, overexpression of similar amount of *GR-1Cα* vectors yielded significantly lower levels of both *GRα* mRNA and protein in DEX-resistant cells compared to DEX-sensitive cells (Fig. 2B). This finding suggests that DEX-resistant cells have stronger negative autoregulatory mechanisms on *GR* expression compared to DEX-sensitive cells.

We then investigated the role of increased expression of *GRα* in endothelial phenotype of both DEX-sensitive and DEX-resistant cells (Fig. 2C, D and E). We chose to study *NOS3* and *SERPINE1* expression, as these genes are key endothelial genes regulated by glucocorticoids, and their expression changes also regulate endothelial phenotype (Lou *et al.* 2001, Muzaffar *et al.* 2005, Kimura *et al.* 2009). *GRα* overexpression correlated with significant decreases in *NOS3* (Fig. 2C) and increases in *SERPINE1* (Fig. 2D) basal mRNA expression in both DEX-sensitive and DEX-resistant cells. Overexpression of *GRα* also increased the basal procoagulant activity of HUVECs as shown by the aPTT assay (Fig. 2E). However, there were significant differences between the sensitivity groups in response to DEX. DEX-treatment led to *NOS3* down-regulation, *SERPINE1* upregulation and aPTT increases in *GR*α-overexpressing DEX-sensitive cells, but not in *GR*α-overexpressing DEX-resistant cells (Fig. 2D and E). Therefore, overexpression of *GR-1Cα* alone did not correct the lack of response to DEX in resistant cells. However, *GR-1Cα* overexpression did alter basal endothelial phenotype in both sensitivity groups suggesting important non-ligand dependent effects of GR in human endothelial cells.

We also investigated the effect of *GR-1Cα* over-expression on its own expression. We found that *GR-1Cα* overexpression (alone or in combination with DEX treatment) decreased the expression of *GR* mRNA isoform 1B in DEX-resistant cells, and that of isoform 1H in both sensitivity groups (Fig. 2F). *GR-1Cα* overexpression did not alter DEX effects on *GR* mRNA isoform expression (Fig. 2F).

Finally, we investigated the expression of GR chaperones FKBP51, BAG1 and HSP90 in HUVECs overexpressing GRα. We found that resistant cells that overexpress GRα have significantly higher expression of the GRα inhibitor FKBP51 and higher levels of HSP90 than sensitive cells (Supplementary Figure 1, see section on supplementary data given at the end of this article), and could be a reason for the lack of DEX response in GRα overexpressing resistant cells. Therefore, differences in DEX-sensitivity in our HUVECs can also be accounted by differential regulation of GR chaperones. In summary, these results demonstrate that exogenous upregulation of *GRα* mRNA/protein levels can significantly alter both basal and glucocorticoid-stimulated endothelial phenotype in HUVECs, but does not eliminate the differences in *GR* expression and function regulation between the sensitivity groups.

### Correlation of *GR* 5′UTR mRNA isoform expression with promoter usage

**Figure 3:**
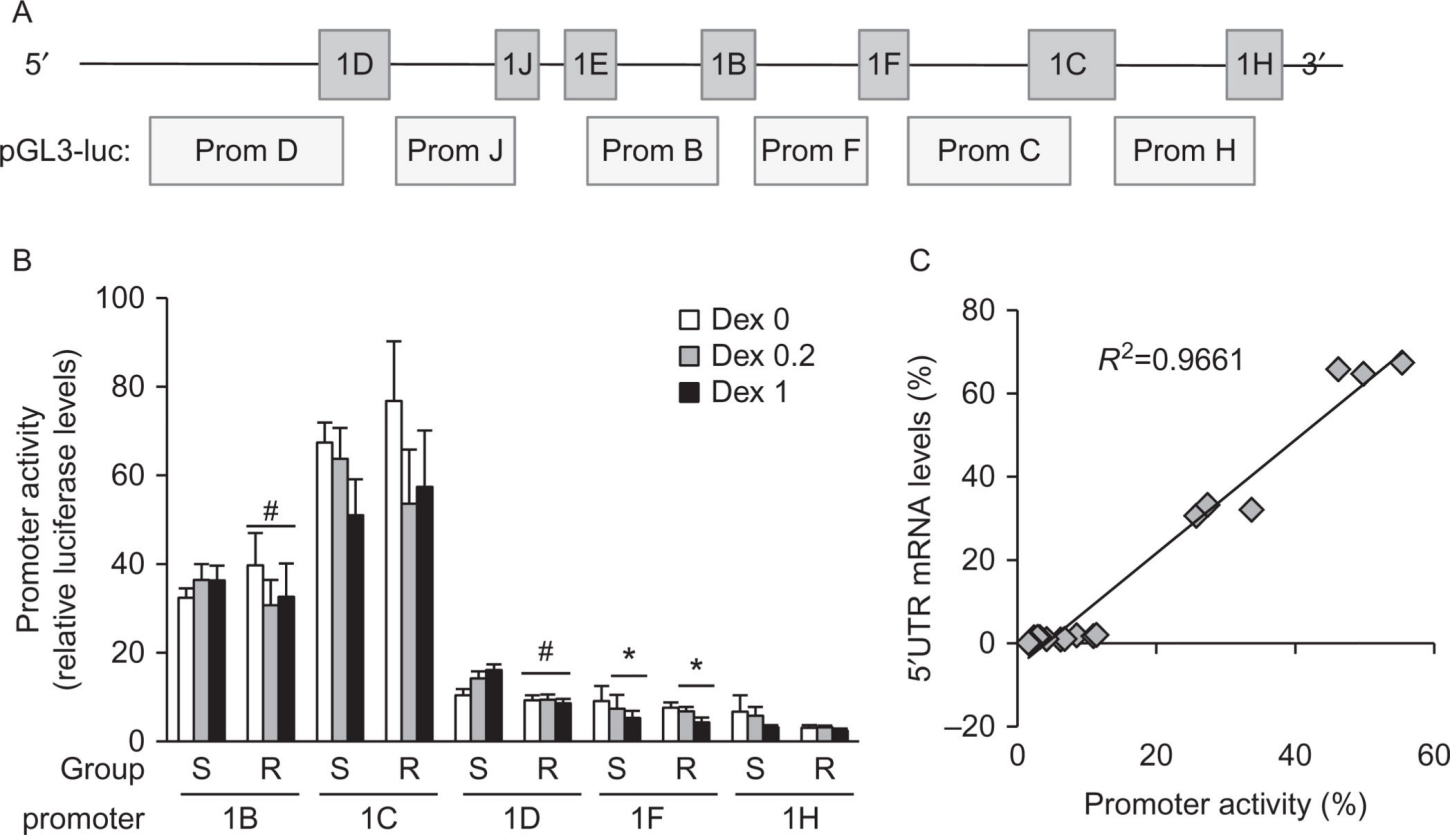
Basal and DEX-stimulated transcriptional activity of proximal *GR* promoters. (A) Structure of the proximal CpG island of the human *GR* gene. (B and C) HUVECs (12 DEX-sensitive and 8 DEX-resistant) were transfected with *GR* promoter-luciferase vectors and then treated with solvent, DEX 0.2, or DEX 1 µM for 24 h. Promoter activity was estimated by the levels of firefly luciferase activity, normalized to Renilla luciferase activity and expressed in fold activity of the empty pGL3 firefly basic reporter vector. Transfections were performed in quadruples. (B) Basal and DEX-dependent *GR* promoter usage in HUVECs. (C) Linear regression analysis of promoter usage (percent luciferase activity) against 5′UTR mRNA isoform expression (percent isoform levels). Averages for all HUVECs for each treatment group (DEX 0, 0.2 and 1 µM) were used. Bars represent the average±s.e.m.. *P<0.05 DEX-treated vs untreated; ^#^*P*<0.05 DEX-resistant cells vs DEX-sensitive cells.

To further characterize the differences observed in *GR* mRNA isoform expression, we cloned five of the proximal TATA-less *GR* promoters to the pGL3 luciferase reporter vector to study promoter activity in HUVECs (Fig. 3A). Clones were sequenced and analyzed for putative internal elements (Inr), DPE, and transcription factor binding sites using the MattInspector software (Supplementary Figure 2, see section on supplementary data given at the end of this article). The consensus Inr element sequence (YYANWYY) located within the first 5 bp of the transcriptional starting site (+1) was found in promoter 1C only. The consensus TATA-less DPE elements (DPE=RGWYV) were found in promoters 1B and 1C, while partial DPEs were found in the remaining *GR* promoters (Supplementary Figure 2). Putative promoter elements for transcription factors were found for each promoter: those reported to be highly expressed in the cardiovascular system are shown in green.

Luciferase assays revealed significant differences in *GR* promoter usage in all HUVECs, with promoter 1C having the highest activity (55%), followed by promoter 1B (30%), promoter 1D (10%), promoter 1F (8%), and promoter 1H (3%) (Fig. 3B). There were no significant differences in basal promoter usage between the sensitivity groups to account for the observed differences in *GR* 5′UTR mRNA isoforms 1C, 1D, and 1F (Fig. 1B). There were only small significant differences in DEX-stimulated promoter usage; sensitive cells responded to DEX with a slight increase in promoter 1B and 1D usage compared to DEX-resistant cells (Fig. 3B). DEX-treatment also significantly decreased promoter 1F usage in both DEX-sensitive and DEX-resistant cells, with non-significant decreases in promoter 1C usage in both groups as well (Fig. 3B). Therefore, our promoter assays suggest that the observed differences in *GR* mRNA isoforms 1C, 1D, and 1F between sensitivity groups (Fig. 1B and C) are not the result of differential transcriptional activation of *GR* promoters.

We analyzed the correlation of promoter usage with 5′UTR mRNA isoform expression by plotting the average % mRNA levels with average % promoter usage in all HUVECs at basal, low DEX (0.2 µM) and high DEX (1 µM) dose (Fig. 3C). We found a significant positive correlation between these two parameters, with only promoter 1D usage being disproportionally higher than the expression of the 1D mRNA isoform (Fig. 3C), suggesting epigenetic regulation of promoter 1D.

### Role of promoter methylation in *GR* 5′UTR mRNA isoform expression

**Figure 4:**
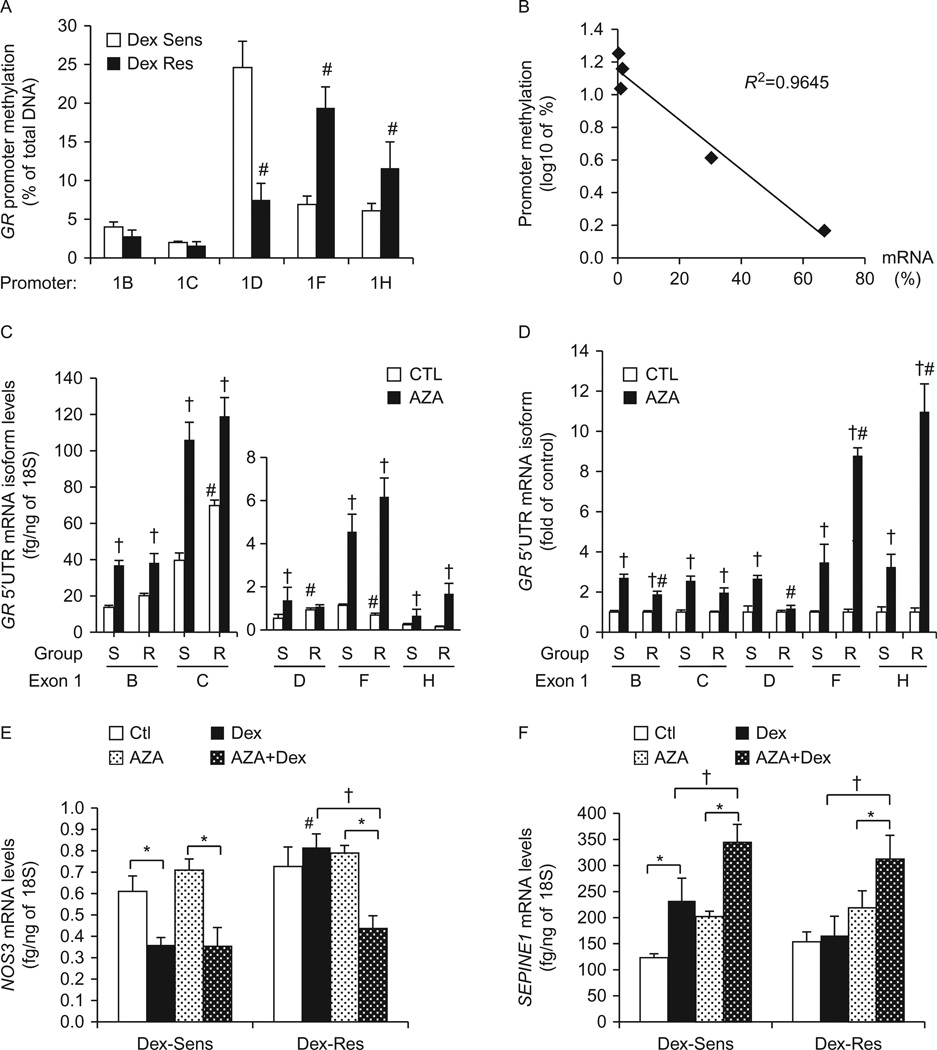
Role of *GR* promoter methylation on *GR* expression and response to DEX. (A) Methylation of proximal promoters as determined by immunoprecipitation of 5-methylcytosine in DEX-sensitive (S) and DEX-resistant (R) cells (*n*=8/group). Results are shown as average percentage methylation. (B) The correlation between basal HUVEC *GR* promoter methylation and *GR* 5′UTR mRNA isoform expression was determined by linear regression analysis. Each data point represents the average levels for each sensitivity group. Promoter methylation percentages are shown as Iog10 values. (C and D) Global demethylation was achieved with 48 h of AZA treatment, and the expression of *GR* 5′UTR mRNA isoform was determined by real-time PCR (*n* = 5/sensitivity group). Results are shown as fg *GR* isoform/ng *18S* RNA (C), and fold expression of untreated cells (D). (E and F) HUVECs were treated with AZA as described for (C) and (D) in the presence or absence of DEX 1µM, and then analyzed for *NOS3* (E) and *SERPINE1* (F) mRNA expression by real-time PCR. Bars represent the average±s.e.m. *P<0.05 DEX-treated vs untreated; ^#^*P*<0.05 DEX-resistant cells vs DEX-sensitive cells; ^†^*P*<0.05 non-AZA vs AZA treatment.

Because our reporter assays failed to reveal transcriptional mechanisms that would account for the observed differences between DEX-sensitive and DEX-resistant cells in *GRα* expression regulation (particularly of mRNA isoforms 1C, 1D, and 1F), we hypothesized that there could be differential methylation patterns of *GR* promoters. We first studied promoter methylation levels by immunoprecipitation of 5-methylcytosine (Fig. 4A). As expected, promoters 1B and 1C showed very low methylation levels of <4 and <1.7% respectively (Fig. 4A). Promoter 1D showed the highest methylation levels of ~20%, followed by promoter 1F (~14%), and promoter 1H (~10%). There were significant differences in promoter methylation levels between DEX-sensitive and DEX-resistant HUVECs (Fig. 4A). DEX-resistant cells had significantly lower methylation levels of promoter 1D and higher methylation levels of promoter 1F, compared to DEX-sensitive cells (Fig. 4A). The basal promoter methylation levels (i.e. averages for all cells: sensitive and resistant) correlated inversely with the expression of *GR* 5′UTR mRNA isoforms, with promoter 1C having the lowest methylation levels and higher mRNA isoform 1C levels, and promoter 1D having the highest methylation levels together with lower mRNA isoform 1D expression levels (Fig. 4B). These results explain why the expression of the 5′UTR mRNA isoform 1D is lower than that of isoform 1F, although the cloned promoter 1D-driven luciferase activity was higher than that of promoter 1F (Figs 1B and 3B), as cloned promoter-vectors do not have any cytosine methylations.

To confirm our MeDIP results, we then investigated the specific methylation sites in promoters 1D and 1F via bisulfite sequencing. Promoters 1D and 1F were chosen because they exhibited the highest methylation levels and because of differences in the expression of mRNA isoforms 1D and 1F between sensitivity groups. Bisulfite sequence analysis confirmed the differences observed in our MeDIP assays (Supplementary Figure 3, see section on supplementary data given at the end of this article and Fig. 4A). We found five methylation sites in promoter 1D with higher methylation percentages in DEX-sensitive cells than DEX-resistant cells (Supplementary Figure 3). DEX-resistant cells showed significant promoter 1F methylation of five different cytosines in comparison with none found in DEX-sensitive cells. In sum, bisulfite analysis confirmed that DEX-sensitive cells have higher methylation levels for promoter 1D but lower methylation levels for promoter 1F, compared to DEX-resistant cells. It is important to note that the MeDIP assay and bisulfite sequencing showed methylation levels for promoter 1F of ~7 and 0%, respectively, in DEX-sensitive cells. This discrepancy is likely due to the presence of other methylation sites further upstream or downstream from the selected DNA segment analyzed via bisulfite sequencing.

Finally, we utilized AZA to induce global demethylation in order to study the role of *GR* promoter methylation on its own expression and function. Analysis of the *GR* 5′UTR mRNA isoform expression demonstrated that AZA treatment resulted in increased expression of all *GR* 5′UTR mRNA isoforms in DEX-sensitive cells, and in all isoforms except isoform 1D in DEX-resistant cells (Fig. 4C and D). Importantly, AZA treatment eliminated the differences in *GR* 5′UTR mRNA isoforms 1C, 1D and 1F expression between DEX-sensitive and DEX-resistant cells (Fig. 4C). Analysis of AZA-induced fold increases in *GR* mRNA expression further highlighted differences between sensitivity groups. AZA-treatment produced higher (fold of control) increases in the expression of *GR* mRNA isoforms 1B and 1D in DEX-sensitive cells compared to DEX resistant cells, and a higher fold increase of isoforms 1F and 1H in DEX-resistant cells compared to DEX sensitive cells, and these data correlated with *GR* promoter methylation levels (Fig. 4A and D).

Consistent with the effect of AZA on *GR* expression, we found that AZA pretreatment increased the response to DEX in the resistant group in terms of *NOS3* and *SERPINE1* gene expression regulation, and thereby eliminated the differential response to DEX between the sensitivity groups (Fig. 4E and F). Of note, AZA treatment significantly increased the basal expression of *SERPINE1* on all cells. This result is likely due to the effect of AZA on *SERPINE1* promoter demethylation and basal transcriptional upregulation. However, we did not study the effect of AZA on the promoter methylation status of other genes. Therefore, AZA effects on *in vitro* DEX response could be mediated by demethylation of other genes that are differentially methylated in DEX-sensitive and DEX-resistant cells. In sum, we found significant differences in *GR* promoter methylation that result in differential *GR* 5′UTR mRNA isoform expression, and these differences further characterize DEX-sensitive and DEX-resistant HUVECs.

## Discussion

We uncovered novel mechanisms of *GR* expression regulation in human endothelial cells that include differential expression of 5′UTR mRNA isoforms due to promoter methylation We have also found significant differences in *GR* promoter methylation and 5′UTR mRNA isoform expression according to DEX-sensitivity. The results of our studies on *GR* mRNA and protein regulation in HUVECs are summarized in Table 1. First, we have determined that the primary 5′UTR mRNA isoform present in human endothelial cells is isoform 1C, followed by isoform 1B, with smaller contributions from isoforms 1F, 1H, and 1D. Previous studies have shown similar dominance of expression of isoform 1C in tissues such as the liver, heart, kidney, and lung (Presul *et al.* 2007, Turner *et al.* 2008, 2010). Our promoter analysis suggests that the presence of putative Inr elements and DPE in promoter 1C leads to transcriptional dominance of mRNA isoform 1C compared to other isoforms. These DNA elements are known to recruit RNA polymerase complexes in TATA-less promoters (Butler & Kadonaga 2002, Yang *et al.* 2007, Juven-Gershon & Kadonaga 2010). Interestingly, mRNA isoforms 1J, 1D, 1H, and 1F have been reported to be highly expressed in non-vascular tissues such as the hippocampus and peripheral mononuclear cells (Turner *et al.* 2008, Cao-Lei *et al.* 2013). In our studies, these exons represent < 10% of all endothelial *GR* transcripts. Therefore, our data indicate that regulation of isoforms 1C and 1B expression, with smaller contributions from mRNA isoforms 1D, 1F and 1H, will have a higher impact on total *GR* mRNA expression.

Previously we reported that increased *in vitro* DEX sensitivity of HUVECs correlated positively with GRα protein levels (Mata-Greenwood *et al.* 2013). DEX-sensitive HUVECs showed lower GRα protein turnover through the proteasome system, and this was partly due to lower expression of the E3 ubiquitin ligase BAG1. In the present study, we have found that increased GRα protein levels observed in DEX-sensitive, compared to DEX-resistant, cells are not the result of increased *GRα* mRNA levels, as previously hypothesized. However, we did observe significant differences in *GRα* mRNA isoform expression among DEX-sensitive and DEX-resistant HUVECs. Resistant cells, but not sensitive cells, responded to DEX treatment with a significant down-regulation of the main *GR* 5′UTR mRNA isoform 1C and 3′UTR mRNA isoform α (Table 1). Because glucocorticoids downregulate *GR*α mRNA expression (Petersen *et al.* 2004, Ramamoorthy & Cidlowski 2013), we now hypothesize that DEX-resistant cells show significant DEX-dependent downregulation of *GR* mRNA expression in addition to increased GR protein degradation via the proteasome. Our *GR*α overexpression data supports this hypothesis; DEX treated-resistant cells had lower levels of overexpressed *GR*α mRNA and protein than DEX-treated-sensitive cells. Therefore, DEX-resistance in endothelial cells could arise from a strong negative autoregulation that includes both mRNA and protein expression decreases.

Another interesting result was that GRα overexpression did not increase *in vitro* response to DEX (measured as changes in *NOS3, SERPINE1*, and aPTT) in resistant cells (Table 1). Furthermore, *GR*α overexpression significantly altered basal endothelial phenotype (*NOS3, SERPINE1*, and aPTT levels) in both sensitivity groups with a greater effect shown in resistant cells. We currently speculate these data to be caused by differences in GR:chaperone interactions between sensitive and resistant cells. In support of this hypothesis, we found that GRα overexpression upregulated the expression of GR inhibitors BAG1 and FKBP51. Further studies on GR chaperones are therefore needed to fully understand the differential *in vitro* response to DEX in HUVECs. In summary, these data suggest that posttranslational regulation of GRα protein, such as GRα proteasomal degradation and GR:chaperone interactions, and not transcriptional mechanisms, are key factors in determining basal and DEX-stimulated levels of biologically active GR and, thereby, in the biological response to glucocorticoids (Table 1).

Perhaps the most novel result of this study is the inverse association between *GR* 5′UTR mRNA isoform expression and *GR* promoter methylation together with the finding of significant differences in promoter methylation between DEX-sensitive and DEX-resistant cells (Table 1). This study is the first to report that methylation plays an important role in *GR* mRNA expression in endothelial cells. DEX-sensitive HUVECs showed significantly higher methylation of promoter 1D, but lower methylation of promoters 1F and 1H. Methylation levels of promoter 1D and 1F correlated inversely with the expression of their corresponding 5′UTR mRNA isoforms 1D and 1F. Of importance was the finding that AZA treatment abrogated the differences in *GR* 5′UTR mRNA isoform expression between sensitivity groups, thereby showing that methylation differences in *GR* promoters are a principal reason for the observed differences in *GR* 5′UTR mRNA isoforms expression between DEX-sensitive and DEX-resistant cells. Unexpectedly, AZA treatment increased the expression of *GR* mRNA isoforms 1B and 1C, even though their proximal promoters were highly unmethylated. We hypothesize that AZA demethylation of upstream sequences, such as promoter 1D or *GR* enhancers, increases the expression of mRNA isoforms 1B and 1C. Our hypothesis is in agreement with previous reports on YY1, a promoter 1D binding transcription factor, and the mediated transcriptional activation of isoform1B (Cao-Lei *et al.* 2011). To our knowledge, this study is the first to show that the expression of *GR* mRNA isoforms 1B and 1C is also regulated by methylation. Numerous studies have shown a significant role of promoter 1F methylation in brain tissue, and its correlation with psychiatric diseases (Moser *et al.* 2007, Alt *et al.* 2010, Labonte *et al.* 2014, Van der Knaap *et al.* 2014). In rats, maternal care has shown to decrease methylation levels of promoter 1–7 in brain and liver tissue (Szyf *et al.* 2005, Witzmann *et al.* 2012), while hypoxia and other stressors increase it (Xiong & Zhang 2013, Gonzalez-Rodriguez *et al.* 2014). Recent human studies on promoter 1F methylation in umbilical cord mononuclear cells revealed increased promoter 1F methylation in gestational infants exposed to maternal stressors (Filiberto *et al.* 2011, Drake *et al.* 2012, Sinclair *et al.* 2012, Hompes *et al.* 2013). This finding is of interest because our DEX-sensitive cells originated from mothers with significantly higher pre-pregnancy BMI than those from the DEX-resistant group. Therefore, we hypothesize that maternal weight could be an important factor (stressor) in determining fetal endothelial glucocorticoid sensitivity. Altogether, our data suggest that differential methylation of *GR* promoters have significance in regulating *GR* mRNA isoform expression and are associated with glucocorticoid sensitivity.

Together, our findings show that *GRα* gene expression in human endothelial cells is highly complex and that *GR* promoter methylation patterns differ between DEX-sensitive and DEX-resistant HUVECs. Although these differences in *GR* promoter methylation and 5′UTR mRNA expression do not account for the differences observed in GRα protein expression, function, and further alterations of endothelial phenotype, we hypothesize that they could be exploited to develop epigenetic markers of vascular glucocorticoid sensitivity. Importantly, global demethylation studies with AZA eliminated the differences between sensitivity groups in the *in vitro* response to DEX, indicating an important role of methylation patterns in establishing HUVEC sensitivity to glucocorticoids. Future research into the mechanisms that lead to differential methylation of *GR* proximal promoters, and the potential role of maternal obesity in programming fetal endothelial glucocorticoid sensitivity, are warranted.

## Supplementary Material

01

## Figures and Tables

**Table 1:** Summary of major findings on GR expression regulation in HUVECs

	
Figure 1: *GR* mRNA isoform levels	Resistant cells have higher expression of *GR* isoforms 1C and 1D, and lower expression of isoform 1F than sensitive cells in basal conditions.
	Dexamethasone-treatment downregulated 5′UTR mRNA isoform 1C and 3′UTR mRNA isoform α in resistant cells only.
Figure 2: Effect of *GR-1Cα* Overexpression	*GR-1Cα* overexpression in resistant cells did not improve *in vitro* response to dexamethasone as determined by *NOS3* downregulation, *SERPINE1* upregulation, and decreases in aPTT.
	*GR-1Cα* overexpression further downregulated the expression of 5′UTR mRNA isoforms 1B (in resistant cells only) and 1H in both sensitivity groups.
Figure 3: *GR* promoter activity	There were no significant differences in basal *GR* promoter (unmethylated) activity between sensitive and resistant cells.
	Dexamethasone treatment decreased promoter 1F activity in both sensitivity groups, decreased promoter 1B activity in resistant cells only and increased promoter 1D activity in sensitive cells only.
	Promoter activity differences between sensitive and resistant cells did not correlate with differences in *GR* 5′UTR mRNA levels.
Figure 4: *GR* promoter methylation and AZA treatment	Sensitive cells have higher promoter 1D methylation levels and lower promoter 1F and 1H methylation levels compared to resistant cells.
	Promoter methylation levels correlated with *GR* 5′UTR mRNA levels.
	AZA treatment upregulated the expression of all *GR* mRNA isoforms except isoform 1D in resistant cells.
	AZA treatment improved *in vitro* dexamethasone response in resistant cells as determined by *NOS3* downregulation and *SERPINE1* upregulation.
	
