# Supplementary material for: Endothelial glucocorticoid receptor promoter methylation according to dexamethasone sensitivity
Source: J Mol Endocrinol. Author manuscript; Available in PMC 2016 Nov 17. (PMC5113289; doi:10.1530/JME-15-0124)
Supplement: 01 [file NIHMS828823-supplement-01.pdf]

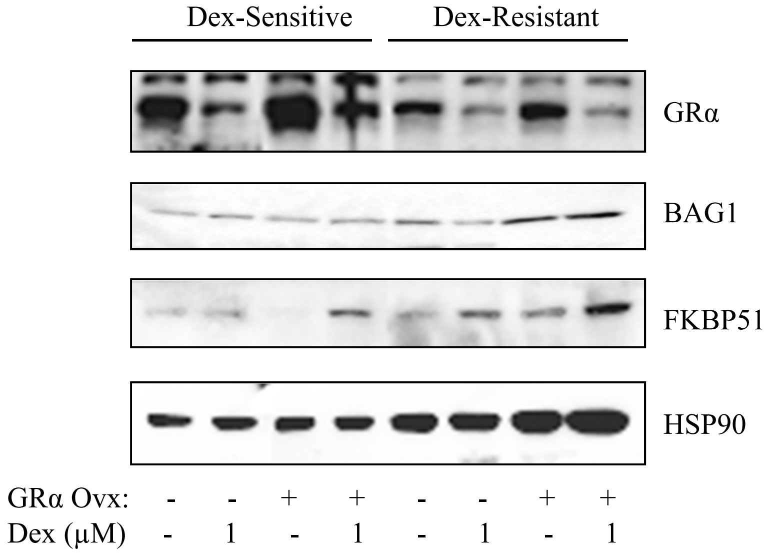

Supplementary Figure 1: Effect of GRα overexpression on GR chaperone expression. HUVECs (3 DEX-sensitive, and 3- DEX-resistant) were transfected with a *GR-1Cα* vector to overexpress GRα, followed by starvation and DEX (1μM) treatment for 24 h. Total protein lysates were prepared and analyzed by immunoblotting for the expression of the GR chaperones FKBP51, BAG1 and HSP90. Representative immunoblots are shown.

GR Proximal Promoter*

Promoter D

-5140 STAT6/RBJK -5120 GSH2/BARX2 -5100 VMYB
TGGGTCTTGATTTGTTTCCCATGAAACTGTTGGTAGTTTAGGGGCCAAATTAACGAAAGA

-5080 -5060 NFAT/VMYB -5040 GRE
CATCTCATTCTAGTGCTTGAGGCTCAGAAATTGGAAAAACGGGCAACTAGGTCAAGCAGT

GRE -5020 -5000 -4980 NF1
GTTCTCATAGCCTCACAGATGAGCATCCAAAGCAAGGGCCTCCTTCTAGTTGACTTGTGC

-4960 -4940 -4920 YY2
CAAGGGACAGGAGAAGCGGAGTCTGCCTTCTGGGTCCAGAAGGGGGTTTTGTTAACATGG

-4900 CDX1 -4880 HSF2 -4860 CMYB
AGTTGCTCAGCGCCCTTCATAAAAATTCTTCTGGCTGAGGGTTCTACGTTGGCATACGGT

CMYB -4840 -4820 ETS1 YY2 -4800 HSF2
TGGTTCCCTCTTCTTTTCCGAGGTGGCGAGTATCTCTTCCTTTGCCAAGATGGCGGCTCC

HSF2 -4780 -4760 -4740
AGAATCCTCTGGAGGCGGCCCCCGTAGATCGTCTCCGGACAAGAGGCTTGCTGAAAGCCT

NFAT -4720 -4700 VMYB HMX2 -4680
ACTTCTTTCCTTTCACATCAGACAATGCACAGGGAACCGTTTACCCTTGAGAACCAAGGA

-4660 -4640 YY1 -4620 MZF1
AGGACGGCTTAGGCTACCCGCGATCGCGAACCTTTGCCAAGATGGTGGCCGCGGGGACGG

-4600 YY1 -4580 ELK1 STAT3 WHN -4560 MZF1
GCTGGCGACACTGTACCCTACCAAGATGGCGGCGGGCGGCTTCCGGGACGCGCTTCCCCA

NFY TGIF -4540 DREAM -4520 -4500 MAZ
ATCGTCTTCAAGATGTCAGAGCAGGGGGAGCCGCCGTCAGTCTGAGCGCGGCGGGAGGTG

-4480 -4460 -4440
AGAGAGTGGCTGTGGCCGAGCGCCCGAGCAGGATTAGGTGGAGCTGCGGCAGCCCCCGCC

Exon 1D

-4420 -4400 -4380
CGTGTCAGGAGCTGGCAAGCGATGTCACCTGTGGGGGCGCAAAAGTTACCTCCCCAAACC

-4360 -4340 -4320
CTAAACCCACACAGCACAACCTTTCCCAGAGTCACAAAAATCATAATCTGTGCCGCACAA

Promoter J

-4300 -4280 SPI1/KLF7/SP1 -4260
GGTAGGAGGCTCGGTCCCGGCATCGTCCAAGCCTTCCCGACGCGGCGAGCTGGGGAAGGG

E2F4 NFKB -4240 HELT -4220 MAZ -4200
AGCTGGGGCGGGGGCTTCCCGCACGGGCACCCCTCGCCCCACGGCCCTCTCCTTTCTCAG

-4180 SPI1/MZF1 -4160 SPI1/MZF1 NFKB -4140 TAL1
GACGGACCACGAGTTCCCTTCCCCTTGGACTGAGGGGGAAGCTCCTAACAGGAACATCTG

BTEB3 -4120 -4100 FOXP1 KLF6 -4080 SP1
TAGGGAGTTGAACGCTGGCATTTTAAAGCTGCCTGTATTTTGTTTTATTTGTAGGGGCAG

ARNT -4060 GATA1 -4040 MESP1 -4020
GGGTCCTATGAACGTGATAGGGTGAGCAACGCACAGAGTCGAGGGCAGCAAATGTCAAGA

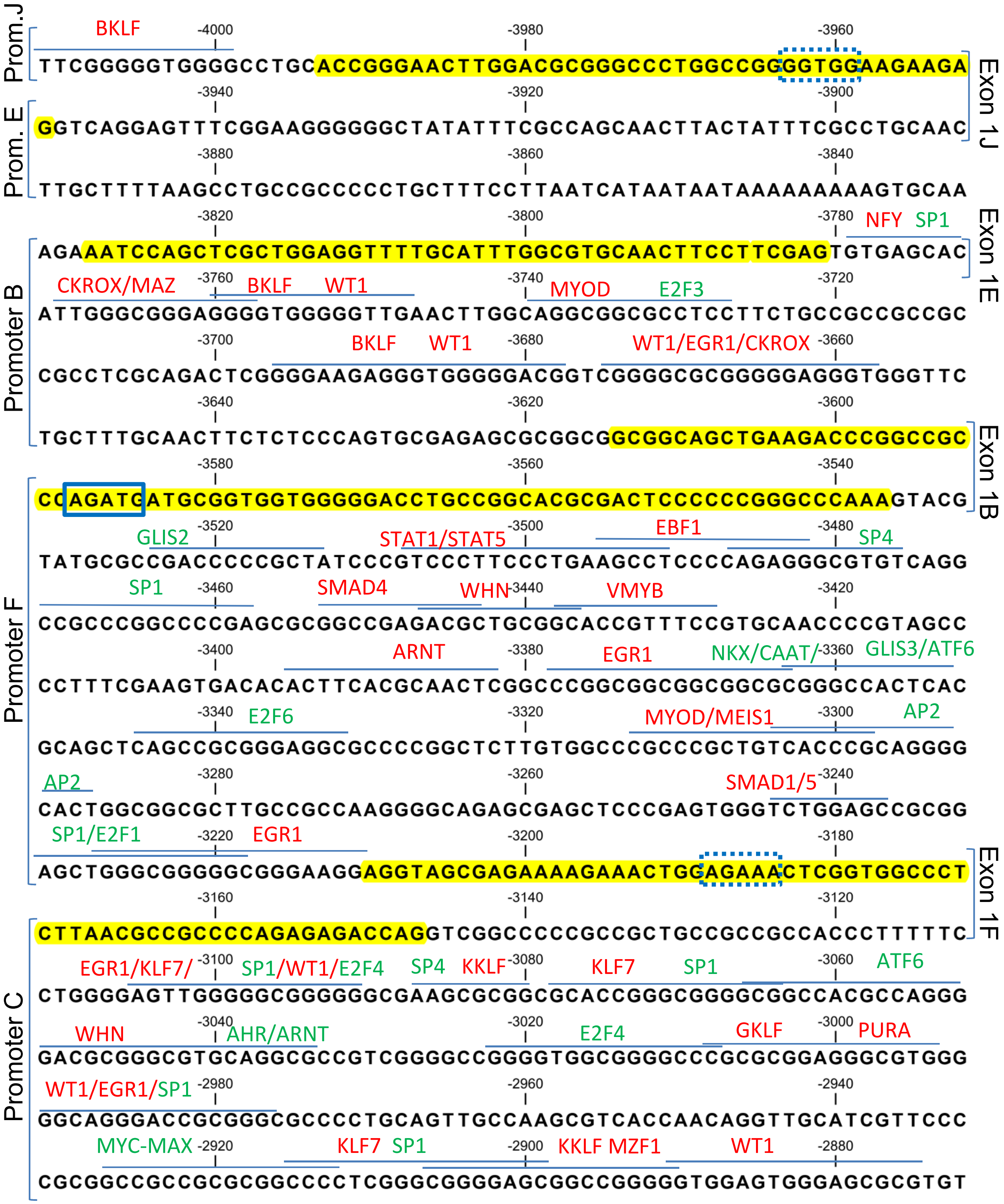
Prom.J
Prom. E
Promoter B
Promoter F
Promoter C
Exon 1J
Exon 1E
Exon 1B
Exon 1F
BKLF -4000 -3980 -3960
TTCGGGGGTGGGGCCTGCACCGGGAACTTGGACGCGGGCCCTGGCCGGGGTGGAAGAAGA
-3940 -3920 -3900
GGTCAGGAGTTTCGGAAGGGGGGCTATATTTCGCCAGCAACTTACTATTTCGCCTGCAAC
-3880 -3860 -3840
TTGCTTTTAAGCCTGCCGCCCCCTGCTTTCCTTAATCATAATAATAAAAAAAAAGTGCAA
-3820 -3800 -3780 NFY SP1
AGAAATCCAGCTCGCTGGAGGTTTTGCATTTGGCGTGCAACTTCCTTCGAGTGTGAGCAC
CKROX/MAZ -3760 BKLF WT1 -3740 MYOD E2F3 -3720
ATTGGGCGGGAGGGGTGGGGGTTGAACTTGGCAGGCGGCGCCTCCTTCTGCCGCCGCCGC
-3700 BKLF WT1 -3680 WT1/EGR1/CKROX -3660
CGCCTCGCAGACTCGGGGAAGAGGGTGGGGGACGGTCGGGGCGCGGGGGAGGGTGGGTTC
-3640 -3620 -3600
TGCTTTGCAACTTCTCTCCCAGTGCGAGAGCGCGGCGGCGGCAGCTGAAGACCCGGCCGC
-3580 -3560 -3540
CCAGATGATGCGGTGGTGGGGGACCTGCCGGCACGCGACTCCCCCCGGGCCCAAAGTACG
GLIS2 -3520 STAT1/STAT5 -3500 EBF1 -3480 SP4
TATGCGCCGACCCCCGCTATCCCGTCCCTTCCCTGAAGCCTCCCCAGAGGGCGTGTCAGG
SP1 -3460 SMAD4 WHN -3440 VMYB -3420
CCGCCCGGCCCCGAGCGCGGCCGAGACGCTGCGGCACCGTTTCCGTGCAACCCCGTAGCC
-3400 ARNT -3380 EGR1 NKX/CAAT/ -3360 GLIS3/ATF6
CCTTTCGAAGTGACACACTTCACGCAACTCGGCCCGGCGGCGGCGGCGCGGGCCACTCAC
-3340 E2F6 -3320 MYOD/MEIS1 -3300 AP2
GCAGCTCAGCCGCGGGAGGCGCCCCGGCTCTTGTGGCCCGCCCGCTGTCACCCGCAGGGG
AP2 -3280 -3260 SMAD1/5 -3240
CACTGGCGGCGCTTGCCGCCAAGGGGCAGAGCGAGCTCCCGAGTGGGTCTGGAGCCGCGG
SP1/E2F1 -3220 EGR1 -3200 -3180
AGCTGGGCGGGGGCGGGAAGGAGGTAGCGAGAAAAGAAACTGGAGAAACTCGGTGGCCCT
-3160 -3140 -3120
CTTAACGCCGCCCCAGAGAGACCAGGTCGGCCCCCGCCGCTGCCGCCGCCACCCTTTTTC
EGR1/KLF7/ -3100 SP1/WT1/E2F4 SP4 KKLF -3080 KLF7 SP1 -3060 ATF6
CTGGGGAGTTGGGGGCGGGGGGCGAAGCGCGGCGCACCGGGCGGGGCGGCCACGCCAGGG
WHN -3040 AHR/ARNT -3020 E2F4 GKLF -3000 PURA
GACGCGGGCGTGCAGGCGCCGTCGGGGCCGGGGTGGCGGGGCCCGCGCGGAGGGCGTGGG
WT1/EGR1/SP1 -2980 -2960 -2940
GGCAGGGACCGCGGGCGCCCCTGCAGTTGCCAAGCGTCACCAACAGGTTGCATCGTTCCC
MYC-MAX -2920 KLF7 SP1 -2900 KKLF MZF1 WT1 -2880
CGCGGCCGCCGCGCGGCCCCTCGGGCGGGGAGCGGCCGGGGGTGGAGTGGGAGCGCGTGT

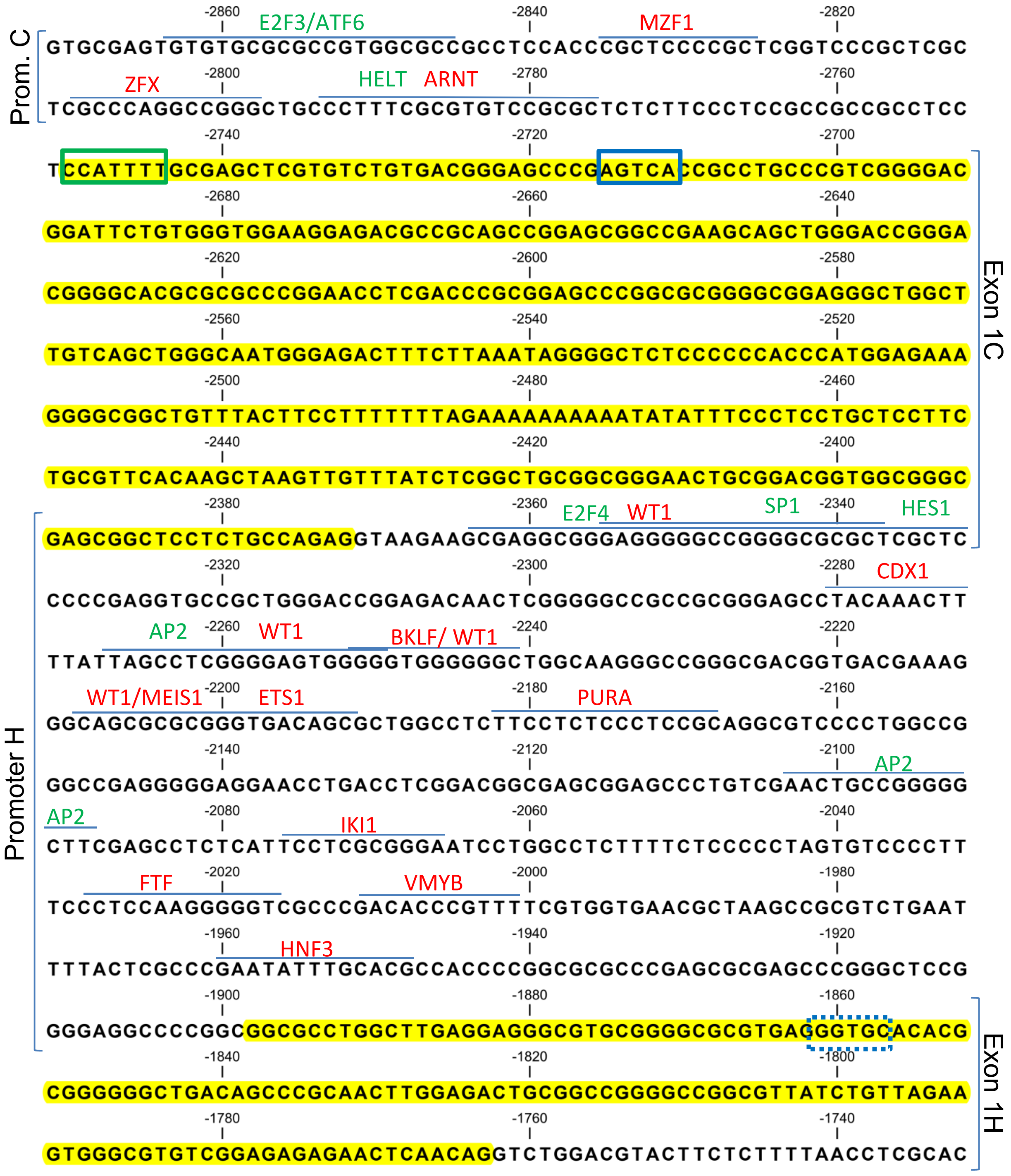
Prom. C
-2860
E2F3/ATF6
-2840
MZF1
-2820
GTGCGAGTGTGTGCGCGCCGTGGCGCCGCCTCCACCCGCTCCCCGCTCGGTCCCGCTCGC
ZFX
-2800
HELT
ARNT
-2780
-2760
TCGCCCAGGCCGGGCTGCCCTTTCGCGTGTCCGCGCTCTCTTCCCTCCGCCGCCGCCTCC
-2740
-2720
-2700
TCCATTTTGCGAGCTCGTGTCTGTGACGGGAGCCCGAGTCACCGCCTGCCCGTCGGGGAC
-2680
-2660
-2640
GGATTCTGTGGGTGGAAGGAGACGCCGCAGCCGGAGCGGCCGAAGCAGCTGGGACCGGGA
-2620
-2600
-2580
CGGGGCACGCGCGCCCGGAACCTCGACCCGCGGAGCCCGGCGCGGGGCGGAGGGCTGGCT
-2560
-2540
-2520
TGTCAGCTGGGCAATGGGAGACTTTCTTAAATAGGGGCTCTCCCCCCACCCATGGAGAAA
-2500
-2480
-2460
GGGGCGGCTGTTTACTTCCTTTTTTTAGAAAAAAAAAATATATTTCCCTCCTGCTCCTTC
-2440
-2420
-2400
TGCGTTCACAAGCTAAGTTGTTTATCTCGGCTGCGGCGGGAACTGCGGACGGTGGCGGGC
Exon 1C
-2380
-2360
E2F4
WT1
SP1
-2340
HES1
GAGCGGCTCCTCTGCCAGAGGTAAGAAGCGAGGCGGGAGGGGGCCGGGGCGCGCTCGCTC
-2320
-2300
-2280
CDX1
CCCCGAGGTGCCGCTGGGACCGGAGACAACTCGGGGGCCGCCGCGGGAGCCTACAAACTT
AP2
-2260
WT1
BKLF/ WT1
-2240
-2220
TTATTAGCCTCGGGGAGTGGGGGTGGGGGGCTGGCAAGGGCCGGGCGACGGTGACGAAAG
WT1/MEIS1
-2200
ETS1
-2180
PURA
-2160
GGCAGCGCGCGGGTGACAGCGCTGGCCTCTTCCTCTCCCTCCGCAGGCGTCCCCTGGCCG
-2140
-2120
-2100
AP2
GGCCGAGGGGGAGGAACCTGACCTCGGACGGCGAGCGGAGCCCTGTCGAACTGCCGGGGG
AP2
-2080
IKI1
-2060
-2040
CTTCGAGCCTCTCATTCCTCGCGGGAATCCTGGCCTCTTTTCTCCCCCTAGTGTCCCCTT
FTF
-2020
VMYB
-2000
-1980
TCCCTCCAAGGGGGTCGCCCGACACCCGTTTTCGTGGTGAACGCTAAGCCGCGTCTGAAT
-1960
HNF3
-1940
-1920
TTTACTCGCCCGAATATTTGCACGCCACCCCGGCGCGCCCGAGCGCGAGCCCGGGCTCCG
Promoter H
-1900
-1880
-1860
GGGAGGCCCCGGCGGCGCCTGGCTTGAGGAGGGCGTGCGGGGCGCGTGAGGGTGCACACG
-1840
-1820
-1800
CGGGGGGCTGACAGCCCGCAACTTGGAGACTGCGGCCGGGGCCGGCGTTATCTGTTAGAA
-1780
-1760
-1740
GTGGGCGTGTCGGAGAGAGAACTCAACAGGTCTGGACGTACTTCTCTTTTAACCTCGCAC
Exon 1H

-1720 -1700 -1680
TTTTTTCTCTTCTCCACCCCCGCCCCGCAAGGGCTTGCTCTTTAGCGTTTGTTGTTAATT

-1660 -1640 -1620
CGCGCCTGAGGTTTCTAAGTGGCCCCTTTTAGAAAAAGACCCCCTGTAACCGTAATGGTT

-1600 -1580 -1560
TTGTGCTGCGATTTTTACAAGTGCTAGTTTGACGTTTGGGGTTGCAGACTTGATAATTGC

-1540 -1520 -1500
AACCTTGTAATACCACTTAAGACCCTCTGGCATGGTTCATTAGGGCCAATTAATGTGGCT

-1480 -1460 -1440
GGGTTATTTGCAACTTAAACTGGGGGATAATGTCGCTTGAGGGAGCGTTTTCGTTTTAGG

-1420 -1400 -1380
AAATATTGTTTTGGTTTCGGGTTTGAAGGCAGCTGTCAAAAAAGCGGCATGGAAATTCAT

-1360 -1340 -1320
TGGGCTCCATTCGATACCTCGTGTTTAGAGATCGTTATCGCCTCAGATAAACGGGGCAGA

-1300 -1280 -1260
GAGGTGGGGAGATAAGCAGTTTACCCTCAAGATTTGTAGTGGCAAGTCCACACCCCTCTC

-1240 -1220 -1200
TCTACCTTCATATTCACTTTTCAGTGAGGGCCAGTGACATTTATGCTGCCTAACGTCATC

-1180 -1160 -1140
GCATAGGAAAAGTTACCTTTTATTGGACGGGATTTGACTATAGTGTCCCAAATGCGCTTC

-1120 -1100 -1080
TCCGTCTTAGCCCATCTCTTAAAACACCCTGATTAACGATATACTAACAGTCTTACTCTC

-1060 -1040 -1020
TTGAGAATAGGCTGAGAATTGGGATAGGTGAAGGTTTGGATAGGTGAAGGCAGAGAAAAT

-1000 -980 -960
TATTTTGAACATTTTACTGGATACAGTTGTACCTGAATTTATATGAATGTGATTTTACGG

-940 -920 -900
TTCTGTGTTTTTCCATTTTTCAGTACTTCGATATTTGTTTGGAAAGGAAAGAACTTAGAG

-880 -860 -840
ATGTAATAGCATTTCATATTGAGGATCTCAAGCAATGTAAACAAATGTAGCTTAATCTAG

-820 -800 -780
ATGTTTTTGTGAGTTATGATAAGGGTCAGCTATATTTAAGTTATGTAAGCTAACAACGTA

-760 -740 -720
GTGAGAAACTACTACACCTTCTCTTCTGCTCTTTAAAATCTAAATTTTAGTTGGCCTATA

-700 -680 -660
TAAAGTGTATCTCATTTCATATATCCAAAATTTGGAGGTAGGCACATCCAGTCAGAAGTA

-640 -620 -600
TGGGTTAAAAAGCCTTTTCCCAGCCTGTCGGAAGATAAGCAGATCAGCATTGTTTATTTT

Intron 1

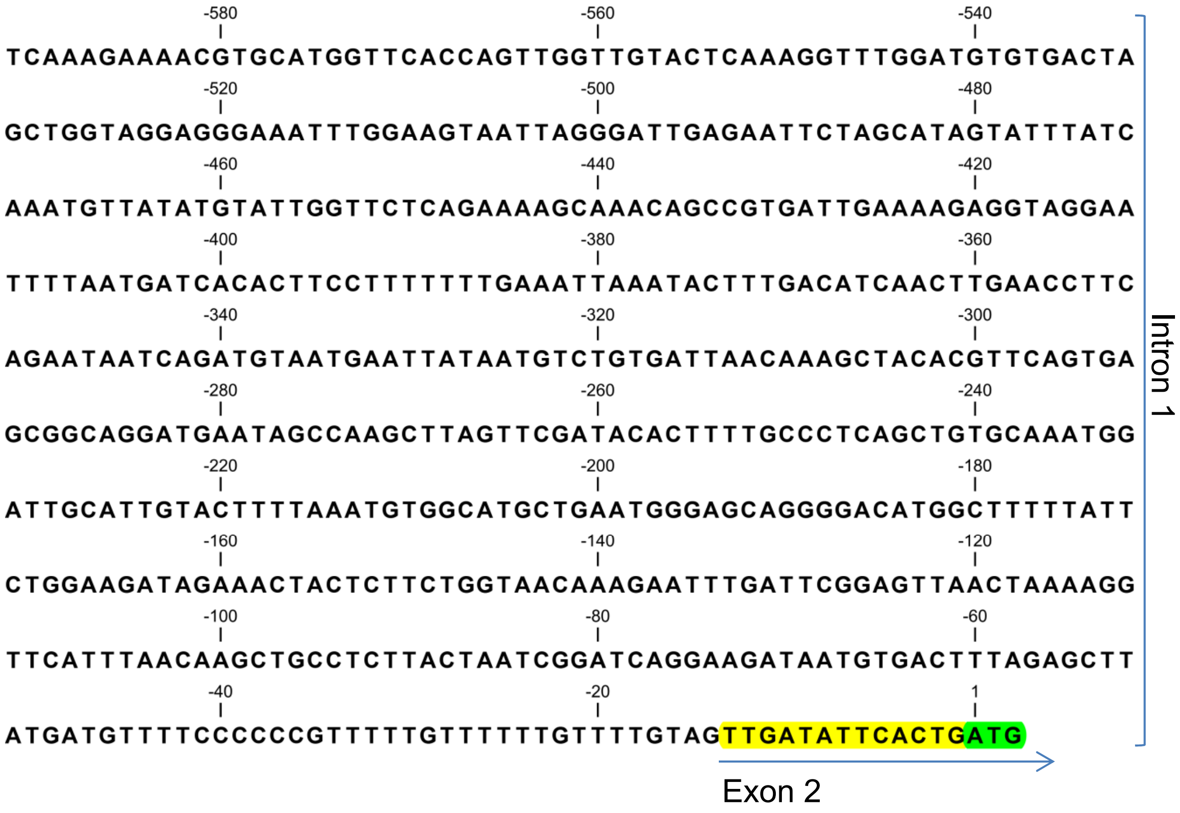

Supplementary Figure 2. The *GR* proximal promoter is shown with its 7 untranslated exons 1 (highlighted in yellow) followed by intron 1 and exon 2 (translational starting point highlighted in green). Analysis of the *GR* proximal promoter was performed with the aid of MattInspector (Genomatix Inc.). Putative Initiator elements (Inr) are shown in green squares and the putative downstream promoters (DPE) are shown in blue squares (dashed squares represent partial sequences instead of consensus sequences). Putative transcription factor elements with a matrix/core similarity higher than 0.9 were selected; transcription factors known to be expressed in the cardiovascular system are shown in green, and the remainder in red.

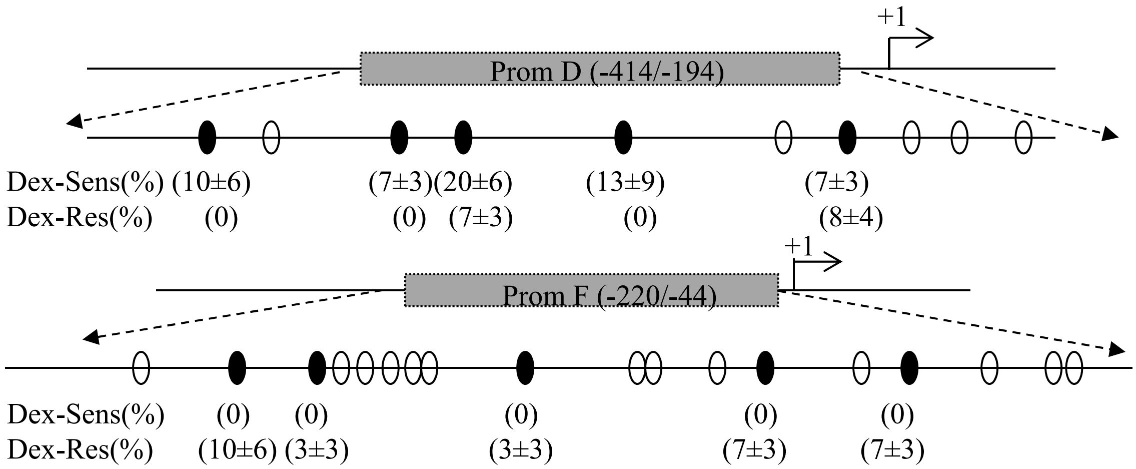

Supplementary Figure 3: Bisulfite sequencing of *GR* promoters 1D and 1F. Dex-sensitive (n=3-4) and Dex-resistant (n=3-4) HUVEC DNA was treated with bisulfite , PCR-amplified, and sequenced as described under methods. Each oval represents one CpG, black ovals represent methylated CpGs. The average ± standard errors for the methylation percentages is shown below.

Supplementary Table 1. Clinical Characteristics of the Study Subjects

	Dex-Sensitive (n=15)	Dex-Resistant (n=10)	*P*
Maternal age, y	31.5 ± 2.0	30.4 ± 2.3	0.701
Pre-pregnancy BMI, kg/m^2	27.2 ± 1.1	22.0 ± 1.3	0.038*
Maternal weight gain, lb	31.8 ± 4.2	37.5 ± 3.8	0.450
Systolic blood pressure, mm Hg	115.8± 2.0	106.5 ± 3.1	0.025*
Diastolic blood pressure, mm Hg	72.3 ± 1.7	68.3 ± 1.4	0.173
Smoking, yes/no	3/12	0/10	0.190
Gestational age (wk)	39.8 ± 0.3	39.1 ± 0.4	0.080
Newborn sex (F/M)	6/9	4/6	0.558
Race, African American/Caucasian (%)	6/9 (40)	5/5 (50)	0.246
Birth weight (g)	3525 ± 114	3401 ± 181	0.574
Umbilical cord blood cortisol, nmol/L	441 ± 10.2	401 ± 11.1	0.061

*p<0.05, Dex-sensitive vs. Dex-resistant.

Supplementary Table 2[1]. SYBR green PCR primers for *GR* mRNA isoforms

Name	Sequence	Annealing Temperature (ºC)	PCR product size
GR-α	5'-CAAAGAGCTAGGAAAAGCCAT-3'<br>5'-CAATACTCATGGTCTTATCCAA-3'	54	161
GR-β	5'-TCAGTTCCTAAGGACGGTCT-3'<br>5'-ACCACATAACATTTTCATGCAT-3'	50	175
GR-P	5'-TGTTTTGCTCCTGATCTGA-3'<br>5'-CCTTTGTTTCTAGGCCTTC-3'	50	223
1F	5'- AGAAACTCGGTGGCCCTCTTA-3'<br>5'- AAGCACACTGCTGGGGTTTT-3'	57	112
1H*	5'-GGCGTTATCTGTTAGAAGTG-3'<br>5'-ATAGAAGTCCATCACATCTC-3'	48	149
18S	5'-CGGCTACCACATCCAAGGAA-3'<br>5'-GCTGGAATTACCGCGGCT-3'	60	78

[1]PCR settings: 15 min hotstart activation at 94 ºC, 55 cycles of 15 sec at 94 ºC, 20 sec at annealing temperature, and 20 sec extension at 72 ºC. Quantification was set at extension.

*Fluorescence quantification set at 76 °C, instead of 72°C.

Supplementary Table 3. Primers used to clone human *GR* proximal promoters

Name*	Sequence	PCR size	Position**
1B Mlu II<br>Bgl II	5'-AACTTGGACGCGTGCCCTGG-3'<br>5'-CACCGCAAGATCTGGGCGGC-3'	409	-3568/-3977
1C Kpn-<br>Xho I	5'-GGAAGGAGGTACCGAGAAA-3'<br>5'-TCGGCCGCTCGAGCTGCGG-3'	568	-2646/-3214
1D Kpn I<br>Xho I	5'-AACTGTTGGTACCTTAGGGGC-3'<br>5'-TAATCCTGCTCGAGCGCTCGG-3'	672	-4443/-5115
1F Kpn I<br>Xho I	5'-TGGTGGGGTACCTGCCGG-3'<br>5'-CCACCGAGTTTCTCGAGTTTCT-3'	402	-3167/-3569
1H Kpn I<br>Xho I	5'-GCGAGCGGTACCTCTGCC-3'<br>5'-CGCCCAGATCTAACAGAT-3'	607	-1775/-2382

*Name of the *GR* promoter and the restriction enzyme sites used.

**Relative to translational start codon ATG in exon 2

Supplementary Table 4. Primers for MeDIP SYBR green PCR analysis and bisulfite sequencing.

Name	Sequence	Annealing Temperature (ºC)	Location relative to Exon 1's starting site
MeDIP Prom 1B	5'-CGCCCCCTGCTTTCCTTAAT-3'<br>5'-TGTGCTCACACTCGAAGGAA-3'	56	-267 to -156
MeDIP Prom 1C	5'-ACCCTTTTTCCTGGGGAGTT-3'<br>5'-ATGCAACCTGTTGGTGACG-3'	55	-371 to -189
MeDIP Prom 1D	5'-GGGCAACTAGGTCAAGCAGT-3'<br>5'-CGCTGAGCAACTCCATGTTA-3'	55	-567 to -416
MeDIP Prom 1F	5-CTATCCCGTCCCTTCCCTGAA-3'<br>5-TGTCACTTCGAAAGGGGCTAC-3'	56	-305 to -187
MeDIP Prom 1H	5'- CGGGAATCCTGGCCTCTTTT-3'<br>5'- CGTGCAAATATTCGGGCGAG-3'	56	-172 to -50
Bisulfite Prom 1D	5'- AAATTTTTTTGGTTGAGGGTTTTA -3'<br>5'- TCCTTCCTTAATTCTCAAAAATAAAC -3'	40	-413 to -191
Bisulfite Prom 1F	5'- TGTGTAATTTTGTAGTTTTTTTTGA-3'<br>5'- AAAACTCACTCTACCCCTTAACAAC -3'	40	-218 to -42
